# Diseases Admitted under the Care of the Physicians of the Westminster Hospital, from August 24 to October 26, 1801

**Published:** 1801-11

**Authors:** 


					[ 39' 3
Diseases admitted under the Care of the Physicians cf the Westmin-
ster Hospitalj from August 24 to Oftober 26, 18,01.
Continued Fever - - - 28
Intermittents - - - - 3
Pleuritis ----- 7
Cynanche ----- 4
Amenorrhcea - - - - 8
Anafarca 7
Anorexia ----- 2
Afcites ------ 1
Afthenia - - - - 13
Afthma ------ i
Cephalasa ----- 4
Cholera -     - 5
Diarrhoea - - 13
JDyfenteria ----- 1
Dyfpepfia ----- - g
Dyfpnoea ----- 4.
Dyfurja ------ g
Enterodynia - - - - 6
Epilepfia - -N - - - 1
Gaftrodynia ----- 6
Gonorrhoea ----- i_
Hasmatemells - - - - 1
Hasmoptoe ----- z
Hvfteria ----- 2
Jfterus ------ 3
Impetigo 7
Lepra - - - - - I
Leucophlegmafia - - - t _
Leucorrhasa - - - - 4
Lumbago . - - 1
Menorrhagia - - - - %
Obftipatio ----- 7
Palpitatio ----- 1
Paralyfis ----- 3
Pertuffis ------ 4
Phthiiis ------ 2
Pleurodyne ----- 5
Prolapfils - - - - - 3
Pyrofis ------ 1
Rheumatifm - - - 21
Scabies - - - - - - 1
Sciatica ------ 3
Schirrhus - - - - 2
Struma ------ I
Tinea ------- I
Tremor ------ 1
Tuffis ------ 10
Worms ------ 3
Vertigo ------ 2

				

